# The role of the *bla*_KPC_ gene in antimicrobial resistance of *Klebsiella pneumoniae*

**Published:** 2019-08

**Authors:** Atossa Ghasemnejad, Monir Doudi, Nour Amirmozafari

**Affiliations:** 1 Department of Biology, Science and Research Branch, Islamic Azad University, Tehran, Iran; 2 Department of Microbiology, Falavarjan Branch, Islamic Azad University, Isfahan, Iran; 3 Department of Microbiology, Iran University of Medical Sciences, Tehran, Iran

**Keywords:** *bla*_KPC_, *Klebsiella pneumoniae*, Infectious disease, Carbapenemase, Colistin

## Abstract

**Background and Objectives:**

*Klebsiella pneumoniae* isolates that produce *K. pneumoniae* carbapenemase (KPC) have become a grave concern for the treatment of infections. KPC-producing strains are not only able to hydrolyze carbapenems but are also resistant to a variety of β-lactam and non-β-lactam antibiotics. The present study evaluated the prevalence of *bla*_KPC_ in *K. pneumoniae* infections and determined the antimicrobial susceptibility of the isolates.

**Materials and Methods:**

The *K. pneumoniae* isolates were identified by biochemical tests and confirmed by genotyping. The modified Hodge test (MHT) was performed to detect carbapenemases, and antimicrobial susceptibility was determined for all isolates by the disc diffusion method. Also, for MHT-positive isolates, supposed to carbapenemases isolates, broth microdilution method was used to measure the minimum inhibitory concentrations (MICs) of meropenem and colistin.

**Results:**

The *bla*_KPC_ genotypic evaluation revealed that only 5 of 96 isolates carried *bla*_KPC_ genes. Antimicrobial pattern showed that isolates carrying *bla*_KPC_ were resistant to cefepime, ticarcillin/tazobactam, and aztreonam discs. Also, results of broth microdilution method showed that KPC-producing *K. pneumoniae* was resistant to meropenem and colistin, according to the CLSI and EUCAST.

**Conclusion:**

In this study nearly half the isolates showed carbapenemase activity as shown by MHT results, but only few of them were carrying *bla*_KPC_. Thus *bla*_KPC_ gene is not the main cause of resistance spread to carbapenems in Isfahan, Iran.

## INTRODUCTION

*Klebsiella pneumoniae* can infect the urinary tract, respiratory tract, surgical sites, and blood-stream, causing severe diseases such as pneumonia, sepsis, and bacteremia (1). *K. pneumoniae* with multiple drug resistant (MDR) pattern has a high prevalence, and the global emergence and dissemination of *K. pneumoniae* strains resistant to carbapenems has increased (2–5). Carbapenems as another β-lactam structure are recommended as first-line therapy for *Enterobacteriaceae* infections, such as *K. pneumoniae*. Also, carbapenems are increasingly used as the first-line antibiotics for unidentified microorganisms and last-line antibiotics for the treatment of infections (1). Resistance to carbapenems is characterized by one or several mechanisms, including carbapenemase production. Carbapenemases exhibit the highest antibiotic resistance, as they can hydrolyze carbapenem antibiotics and other β-lactam antibiotics (6).

*Klebsiella pneumoniae* carbapenemase (KPC) is one of the main cause of resistance to carbapenems in *K. pneumoniae*, and it can widely spread due to its location on various plasmids (7–10). KPC enzymes constitute a major family of class A serine carbapenemases, which are mainly produced by *K. pneumoniae*. KPC is, in fact, the most common cause of carbapenem resistance in *Enterobacteriaceae* (7, 11, 12). KPC producing *K. pneumoniae* (KPC-Kp) has affected many countries, especially Greece and Italy, and has rapidly become endemic in some countries, such as China, Colombia, and Puerto Rico (13–18). Dissemination of KPC around the world highlights the role of *bla*_KPC_ gene in the spread of antimicrobial resistance; Thus, strains harboring *bla*_KPC_ gene are a major cause of concern for healthcare systems around the world (19). Therefore, in this study, we aimed to evaluate the prevalence and antimicrobial susceptibility of KPC-producing *K. pneumoniae* among patients in Isfahan, Iran.

## MATERIALS AND METHODS

### Bacterial collection.

*K. pneumoniae* isolates were collected from Al-Zahra hospital, Isfahan, Iran between February and June 2016. The clinical specimens included urine samples, wound swabs, peritoneal fluid sample, blood samples, respiratory secretions, and others (20).

### PCR detection of *Klebsiella pneumoniae*.

After biochemical test for detection of isolates, polymerase chain reaction (PCR) based on 16S-23S rDNA internal transcribed spacer (ITS) was performed for the identification of *K. pneumoniae* isolates using specific primers (forward, ATTTGAAGAGGTTGCAAACGAT; reverse, TTCACTCTGAAGTTTTCTTGTGTTC). The procedure was performed as follows. Five colonies from the overnight culture in trypticase soy agar (TSA) medium were suspended in 100 μl distilled water. For DNA extraction, boiling lysis method was used. Cell debris was centrifuged at 13684 RCF for 3 minutes. Supernatants were used as the source of template DNA for amplification. The cycling conditions were as follows; 10 min at 94°C, followed by 35 cycles of 30 s at 94°C, 20 s at 57°C, and 20 s at 72°C, then 10 min hold at 72°C. Ten microliter of the product was detected by 1% (w/v) agarose gel electrophoresis in 1X TAE buffer (20, 21).

### Phenotypic confirmation of KPC.

According to the CLSI guidelines, MHT was performed for the detection of carbapenemases. Due to the low specificity of MHT method, both meropenem and ertapenem discs were used separately as the substrates for MHT (20, 22).

### Genotypic confirmation of KPC.

PCR was run for MHT-positive strains to screen the presence of *bla*_KPC_ gene. *K. pneumoniae* ATCC BAA 1705 and *K. pneumoniae* ATCC BAA 1706 were used as the quality control strains. The forward and reverse primers were KPC-F: ATGTCACTGTATCGCCGTCT and KPC-R: GCTGTGCTTGTCATCCTTGT, respectively. The primers were used at a concentration of 1 μM. PCR was performed as follows. Initial denaturation at 95°C for 5 min, followed by 35 cycles of denaturation at 95°C for 60 s, annealing at 53°C for 30 s, elongation at 72°C for 90 s, and a final extension at 72°C for 10 min. The amplification product was approximately 819 bp in length (20).

### Antimicrobial susceptibility testing.

The susceptibility test was performed based on the disc diffusion method, using β-lactam and non-β-lactam antimicrobial discs (i.e., cefepime 30 μg, ciprofloxacin 5 μg, imipenem 10 μg, meropenem 10 μg, ertapenem 10 μg, ceftazidime 30 μg, gentamicin 10 μg, and trimethoprim-sulfamethoxazole 1.23/25.75 μg), according to CLSI guidelines (22). The quality control strains for the susceptibility test included *Escherichia coli* ATCC 25922, *K. pneumoniae* ATCC 700603, and *Pseudomonas aeruginosa* ATCC 27853. In addition, the minimum inhibitory concentrations (MICs) of meropenem and colistin were determined via broth microdilution for the positive modified Hodge test (MHT) strains as potential carbapenemase producing isolates. The results were interpreted based on the CLSI and European Committee on Antimicrobial Susceptibility Testing (EUCAST) guidelines (22–24).

## RESULTS

### Isolate distribution.

Ninety-six *K. pneumoniae* isolates were isolated from the following departments: intensive care unit (34.38%), emergency ward (17.71%), outpatient unit (15.63%), surgery ward (9.38%), rheumatology and nephrology unit (5.21%), neonatal intensive care unit (4.17%), neurology unit (3.13%), gynecology ward (3.13%), neonatal unit (2.08%), orthopedics (1.04%), laparoscopy unit (1.04%), ear-throat-nose unit (1.04%), Radiology (1.04%), and unknown units (1.04%). Overall, 59 (61.5%) patients were male, and 37 (38.5%) patients were female (20).

### KPC isolates.

A total of 54 isolates showed positive results, 37 isolates were classified as carbapenem-resistant using MHT with the meropenem disc, and 47 with the ertapenem disc (some isolates showed positive result with the use of both discs) (20). Five out of 54 Carbapenemase-producing isolates harbored *bla*_KPC_ genes, as detected by PCR (20).

### Antimicrobial resistance patterns.

The antimicrobial susceptibility test using the disc diffusion method showed that 69 (71.88%) isolates were resistant to cefepime, ciprofloxacin, and imipenem. On the other hand, 60 (62.5%) isolates were resistant to meropenem, 49 (51%) isolates were resistant to ertapenem, 68 (70.83%) isolates were resistant to ceftazidime, 61 (63.54%) isolates were resistant to gentamicin, and 52 (54.17%) isolates were resistant to trimethoprim-sulfamethoxazole.

Disc diffusion assay of MHT-positive isolates showed that the non-producing KPC isolates were highly resistant to carbapemens (Table 1) (20). Also, MHT-positive isolates micro broth dilution for meropenem showed that 7 (13%) isolates were sensitive and 47 (87%) isolates were resistant (Table 2); micro broth dilution for colistin showed that 1 (1.85%) isolate was sensitive and 53 (98.15%) were resistant (Table 3).

**Table 1.** Antimicrobial susceptibility of MHT-positive isolates to Carbapenem discs.

**Carbapenem discs**		**KPC**	

		**Negative**	**Positive**
Imipenem	Resistant	67.7%	4.2%
	Intermediate	15.6%	0.0%
	Sensitive	11.5%	1.0%
Meropenem	Resistant	58.3%	4.2%
	Intermediate	15.6%	1.0%
	Sensitive	20.8%	0.0%
Ertapenem	Resistant	46.9%	4.2%
	Intermediate	11.5%	0.0%
	Sensitive	36.5%	1.0%

**Table 2.** MIC of Meropenem for all MHT-positive isolates. All five isolates that carrying *bla*_KPC_ were resistant to meropenem.

		**MIC colistin**										

		0.125	0.25	0.5	1	2	4	8	16	32	64	>64
KPC	Negative	2	2	2	1	0	1	1	0	4	8	28
	Positive	0	0	0	0	0	0	0	1	1	2	1

**Table 3.** MIC of Colistin for all MHT-positive isolates. All five isolates that carrying *bla*_KPC_ were resistant to colistin.

		**MIC colistin**											

		0.25	0.5	1	2	4	8	16	32	64	128	256	>256
KPC	Negative	0	1	0	0	1	2	10	14	6	2	3	10
	Positive	0	0	0	0	0	0	1	0	2	2	0	0

### Antimicrobial resistance patterns of KPC-*Kp.*

The MICs of meropenem for all 5 KPC-*Kp* were as follows: 1 isolate (16 mg/l), 1 isolate (32 mg/l), 2 isolates (64 mg/l), and 1 isolate (>64 mg/l). Also MICs of colistin against 5 KPC-*Kp* isolates showed resistant, 2 isolates (128 mg/l), 2 isolates (64 mg/l), and 1 isolate (16 mg/l).

Disc diffusion assay for KPC-*Kp* isolates showed that 4 out of 5 isolates were resistant to imipenem, ceftazidime, ciprofloxacin, and gentamicin. All KPC-producing isolates (5 isolates) were resistant to cefepime, ticarcillin-tazobactam, and aztreonam. On the other hand, 3 out of 5 KPC-*Kp* isolates were sensitive to trimethoprim-sulfamethoxazole. One out of 5 KPC-*Kp* isolates was sensitive to gentamicin, whereas 1 out of 5 KPC-*Kp* isolates showed intermediate to ciprofloxacin.

## DISCUSSION

The prevalence of infection with KPC-producing *K. pneumoniae* has generally increased worldwide. Sporadic, epidemic, or endemic isolation of KPC-*Kp* has been reported. While sporadic outbreaks occurred in France, Spain, Germany, Ireland, and Puerto Rico, endemic cases were reported in USA, Colombia, Greece, and Italy (13, 25–28). In fact, KPC-*Kp* strains have become the major MDR pathogens, causing nosocomial infections (27). In this study, molecular epidemiology of *bla*_KPC_ gene in *K. pneumoniae* indicated a low prevalence in Iran.

One study indicated that two of the most effective antibiotic groups for KPC-*Kp* are polymyxins, and aminoglycosides (28). Previous reports indicated that KPC-producing isolates are commonly resistant to many antibiotics such as colistin, and carbapenems (29–31). Also, some studies demonstrated that KPC-producing isolates are susceptible to gentamicin and colistin and intermediate or sensitive to tigecycline (29, 30, 32). However the present findings showed that all KPC-*Kp* isolates were resistant to colistin and that most of the isolates were resistant to gentamicin. Also, KPC-*Kp* showed resistance to imipenem, ceftazidime, ciprofloxacin, gentamicin, cefepime, ticarcillin-tazobactam, and aztreonam discs.

In this study, the disc diffusion assay of all isolates showed that the highest antibiotic resistance was related to imipenem, ciprofloxacin, and cefepime, whereas highest antibiotic susceptibility was related to trimethoprim-sulfamethoxazole. Most of the MHT positive isolates were resistant to colistin. Whereas the MIC of meropenem against 28 MHT positive isolates (non-KPC-producing) exceeded 64 mg/l.

## CONCLUSION

The present study revealed sporadic outbreaks of KPC-producing *K. pneumoniae*. It is important to control the spread of this infection by investigation specific mechanisms of carbapenem resistance and employing effective methods such as active surveillance in health centers (for decreasing health associated infections). Also, the results confirmed the importance of implementing infection control programs to identify and prevent the prevalence of other carbapenemase-producing *K. pneumoniae* rather than only KPC-*Kp*. Due to the antimicrobial patterns of isolates, it is important to find effective options like new antibiotics or antibiotics combinations.
